# Spindle cell carcinoma of the breast as complex cystic lesion: a case report

**DOI:** 10.7497/j.issn.2095-3941.2014.02.008

**Published:** 2014-06

**Authors:** Masahiro Kitada, Satoshi Hayashi, Yoshinari Matsuda, Kei Ishibashi, Keisuke Oikawa, Naoyuki Miyokawa

**Affiliations:** ^1^Department of Surgery, ^2^Department of Clinical Pathology, Asahikawa Medical University, Asahikawa Hokkaido 078-8510, Japan

**Keywords:** Breast cancer, spindle cell carcinoma, cystic lesion

## Abstract

Spindle cell carcinoma of the breast is a rare tumor. This tumor can proliferate rapidly and cause cystic changes because of internal tissue necrosis. We evaluated a 54-year-old woman with right breast lump. Mammography showed a category four mass with a diameter of 2.5 cm. Ultrasonography (US) revealed a complex cystic lesion, and fine-needle aspiration (FNA) cytology demonstrated bloody fluid and malignant cells. Partial breast resection and sentinel lymph node biopsy were performed. Immunohistology revealed spindle cells with positive results for cytokeratin (AE1/AE3) and vimentin, partially positive results for s-100, and negative results for desmin and α-actin. The pathological stage was IIA, and biochemical characterization showed that the tumor was triple negative. Six courses of FEC-100 chemotherapy (5-fluorouracil 500 mg/m^2^, epirubicin 100 mg/m^2^, and cyclophosphamide 500 mg/m^2^) were administered. Radiotherapy was performed. This case is discussed with reference to the literature.

## Introduction

Spindle cell carcinoma of the breast is composed of a mixture of sarcomatoid spindle and epithelial adenocarcinoma cells, representing carcinosarcoma. This entity is now classified as a subtype of malignant epithelial tumor. In this relatively rare histological type, rapid proliferation may cause cystic changes because of internal tissue necrosis. In this paper, we report a rare case of spindle cell carcinoma in breast, and this study was approved by the institutional ethics committee.

## Case report

A 54-year-old woman presented to her personal physician and complained the pain on her right breast. She underwent a breast surgery in our hospital. The patient and her family’s medical histories were unremarkable.

The patient was 157 cm tall and weighed 53 kg. Physical examination of the right breast (area C) revealed an elastic hard mass measuring 2.5 cm × 2.5 cm. Margins were distinct, with relatively good mobility and a tumor-nipple distance of 3.2 cm. No axillary or cervical lymph nodes were palpable. Hematological and biochemical blood test results were normal. The levels of tumor markers, such as carcinoembryonic antigen, carbohydrate antigen 15-3, and National Cancer Center-Stomach-439, were within normal limits.

**Figure 1:**
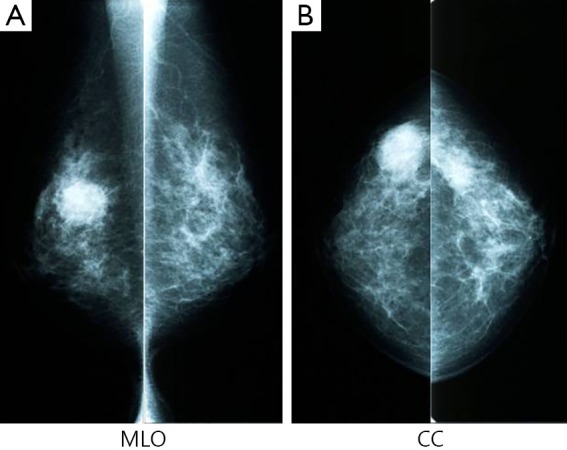
Mammography revealed a round, highly dense 2.5 cm mass with fine serrations on the right-middle outside area (A, MLO; B, CC).

**Figure 2:**
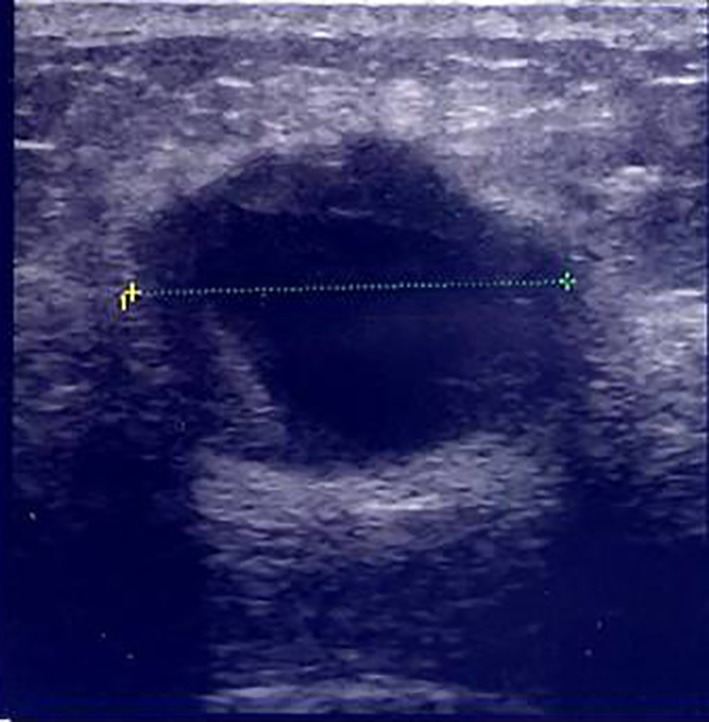
Breast US revealed complex cystic lesion, and the tumor was large with 2.5 cm diameter.

**Figure 3:**
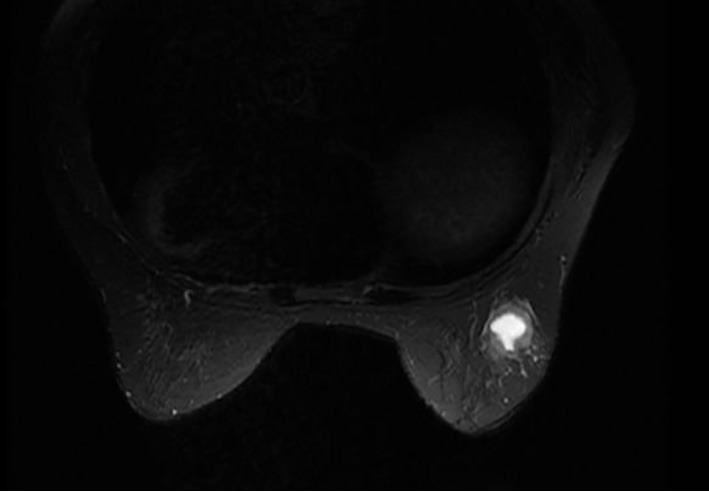
Preopetative breast MRI (T2 weighted image). MRI findings and a T2 weight image revealed an early enhancement image with no intraductal spread. MRI, magnetic resonance imaging.

Mammography revealed an indistinct margined hyper dense mass in the right outer portion, which was assessed as Breast Imaging-Reporting and Data System category 4C (**Figure 1A**: MLO view, **Figure 1B**: CC view). Breast ultrasonography (US) revealed complex cystic lesion, and the tumor was large with 2.5 cm diameter (**Figure 2**). Complex cystic lesion on US with blood content on fine-needle aspiration (FNA) cytology revealed malignant cells. Preoperative magnetic resonance imaging (MRI) findings and a T2 weight image revealed an early enhancement image with no intraductal spread (**Figure 3**). No distant metastases was identified on computed tomography or bone scan. The tumor stage was T_2_N_0_M_0_. Breast-conserving surgery and sentinel lymph node biopsy were performed.

**Figure 4:**
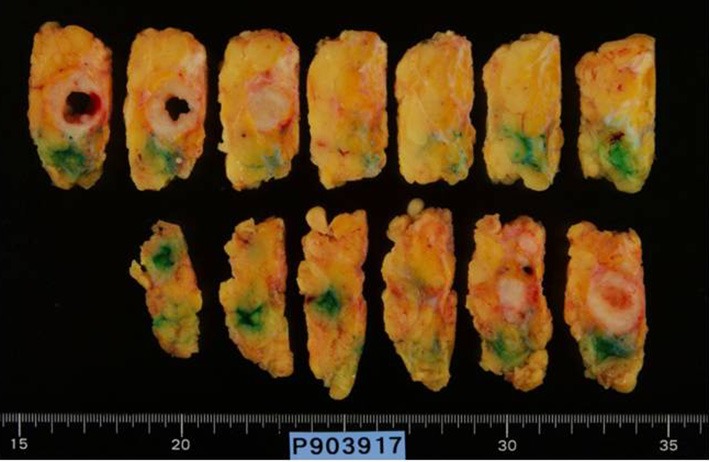
Resected specimen of cut surface; the tumor measured 2.0 cm in diameter and contained a central cystic area.

**Figure 5:**
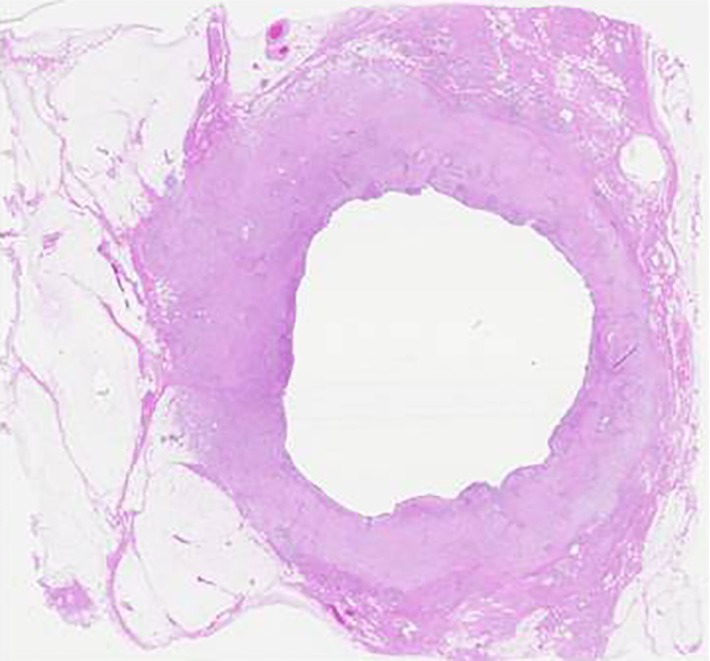
Macroscopic image of the resected specimen; the tumor measured 2.0 cm in diameter and contained a central cystic area.

**Figure 6:**
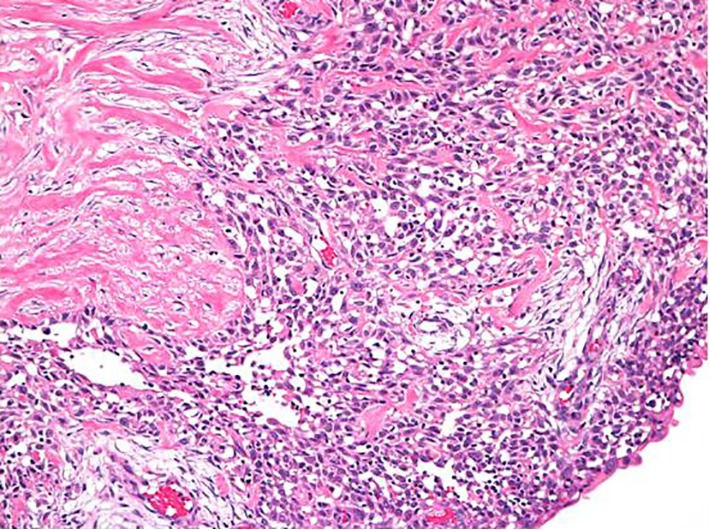
Histopathological finding showed low papillary growth in the cystic cavity surface, which was covered by adenocarcinomatous components associated with some squamous metaplasia. The tumor was diagnosed as spindle cell (H&E staining ×100).

The results exposed two sentinel lymph nodes and no malignant cell. The tumor measured 2.0 cm in diameter and contained a central cystic area (**Figures 4****,****5**). Histopathological examination showed low papillary growth in the cystic cavity surface, which was covered by adenocarcinomatous components associated with some squamous metaplasia. Most of the lesions comprising the cyst wall were a mixture of irregular bundles of spindle-shaped tumor cells and fibrous connective tissue proliferation. The tumor was diagnosed as spindle cell (**Figure 6**).

**Figure 7:**
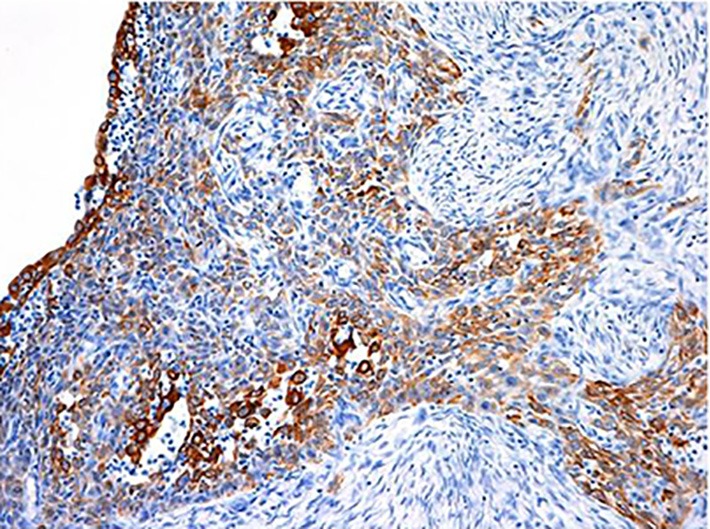
Immunohistological findings of the positive staining for cytokeratin (AE1/AE3) (H&E staining ×100).

**Figure 8:**
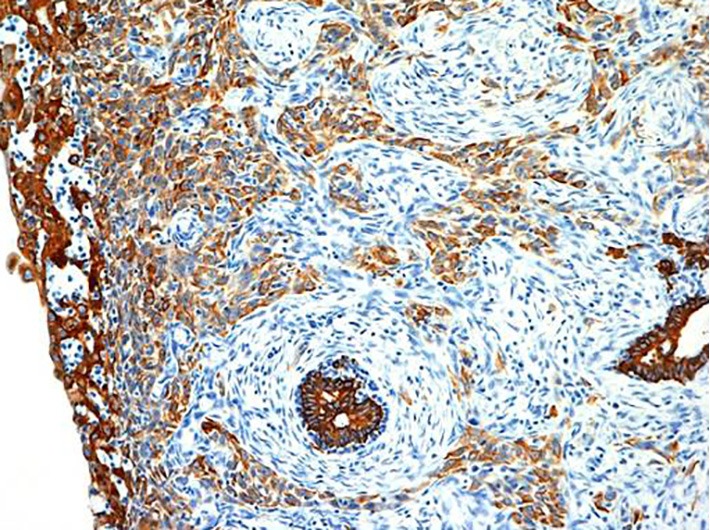
Immunohistological findings of the positive staining for vimentin (H&E staining ×100).

Immunostaining of spindle-shaped tumor cells showed positive results for cytokeratin (AE1/AE3) and vimentin (**Figures 7****,****8**), partially positive results for s-100, and negative results for desmin and α-actin. The final histopathological diagnosis was spindle cell carcinoma. Histological grade from malignancy was grade III. Lymphatic invasion was negative, whereas blood vessel invasion was positive. Lymph node metastasis was negative, and p-stage I was diagnosed. Estrogen receptor, progesterone receptor, and human epidermal growth factor receptor type 2 (HER2), which showed negative results by biochemical tests, were called triple negative type in subtype classification. The postoperative course was uneventful. The patient received six courses of FEC-100 chemotherapy. Radiotherapy included simultaneous integrated boost enforced 60 Gy (2 Gy/day) to the residual breast tissue. After 24 months of postoperation, no recurrence has been detected.

## Discussion

Spindle cell carcinoma of the breast is a rare histological carcinoma, accounting for approximately 0.08% of all breast cancers^1^. Together with squamous cell carcinoma and carcinoma with osseous and chondroid metaplasia, this entity is classified as a metaplastic carcinoma^2^. The tumor comprises spindle-shaped cells with a sarcomatoid appearance, representing carcinosarcoma. In the present case, some tumor cell nests with epithelial features and areas of squamous metaplasia were observed. The sarcomatoid component is defined as a component by which epithelial cancer cells assume a spindle shape. In our hospital, which has been operating for over 10 years, only 3 (0.024%) of 1,280 breast cancer surgery cases have involved spindle cell carcinoma.

At the time of initial diagnosis, numerous spindle cell carcinomas tend to be large and localized, with histopathological examination often showing well-defined margins or a partially nodular appearance^3^. The image view of spindle cell carcinoma reveals a large size and nonspecific shadow, clear of the boundary around on mammography, and high signal intensity on T2 MRI images^4^. On breast US, the cystic change is not rare, these cysts are not true cysts with a capsule, but rather result from internal degeneration and necrosis because of relatively rapid tumor proliferation.

Breast-conserving surgery is often difficult because of the large tumor size in numerous patients^5^. A report has indicated that the tumor average diameter is 4 cm^6^. Lymph node metastasis is less common than other breast cancers^6^^,^^7^. In the present case, breast-conserving surgery is possible when minimal peripheral invasion or intraductal spread is present.

Histopathological examination revealed spindle-shaped sarcomatoid cell proliferation and a distinct area of epithelial cancer cells. In the sarcomatoid component, epithelial characteristics were decreased; therefore, immunostaining revealed a mixture of areas positive for epithelial marker cytokeratin and mesenchymal marker vimentin. Many of the tumors discussed above are histological grade from malignancy grade III, but the prognosis may be poor even in grade I^8^. This finding is largely attributable to biochemical characteristics on the tumor, such as the high negative rates of hormone receptors and HER2/neu protein^6^^,^^9^. Given this triple-negative status exhibited in many cases, hormone and molecular-targeted therapies are often ineffective. Treatment therefore usually consists of chemotherapy. However, no specific chemotherapy regimens has been devised for metaplastic carcinoma, including spindle cell carcinoma. As spindle cell carcinoma shows sarcomatoid features, the efficacy of chemotherapy is also often uncertain^5^. Treatment is thus based on protocols for invasive ductal cancer. In our patient, the histopathological diagnosis was T_2_N_0_, grade III, triple-negative; therefore, six courses of FEC-100 were administered. No sign of recurrence has been identified as of the time of writing, that is, 10-month postoperation. Nevertheless, careful follow-up observation will be required.

## Conclusion

We reported rare spindle cell carcinoma in breast presenting as complex cystic lesion with blood content at FNA. Enforced breast-conserving surgery and sentinel lymph node biopsy were performed.
