# Erratum to spindle cell carcinoma of the breast as complex cystic lesion: a case report

**DOI:** 10.7497/j.issn.2095-3941.2014.03.008

**Published:** 2014-09

**Authors:** Masahiro Kitada, Satoshi Hayashi, Yoshinari Matsuda, Kei Ishibashi, Keisuke Oikawa, Naoyuki Miyokawa

**Affiliations:** ^1^Department of Surgery, ^2^Department of Clinical Pathology, Asahikawa Medical University, Asahikawa Hokkaido 078-8510, Japan

In the published article^1^, two errors appeared on page 132. The legends of **Figure 7** and **Figure 8** should have read as follows:

**Figure 7** Immunohistological findings of the positive staining for cytokeratin (AE1/AE3) (IHC staining, ×100).

**Figure 8** Immunohistological findings of the positive staining for vimentin (IHC staining, ×100).

The authors and the journal apologize for the errors and for any confusion it may have caused.
